# Prevalence and factors associated with neonatal hypothermia on admission to neonatal intensive care units in Southwest Ethiopia – A cross-sectional study

**DOI:** 10.1371/journal.pone.0218020

**Published:** 2019-06-06

**Authors:** Gebresilasea Gendisha Ukke, Ketema Diriba

**Affiliations:** 1 Department of Midwifery, College of Medicine and Health Sciences, Arba Minch University, Arba Minch, Ethiopia; 2 Department of Nursing, College of Medicine and Health Sciences, Arba Minch University, Arba Minch, Ethiopia; Cincinnati Children's Hospital Medical Center, UNITED STATES

## Abstract

**Introduction:**

Neonatal hypothermia is one of the main underlying factors associated with neonatal deaths.

**Objective:**

The objective of this study was to assess the prevalence and factors associated with neonatal hypothermia on admission to neonatal intensive care units in Southwest Ethiopia.

**Methods:**

Institution-based cross-sectional study design was employed between February to September 2017 at intensive care units of Arba Minch and Jinka General Hospitals. All neonates admitted to the two neonatal intensive care units during the study period were included in the study. Data were collected by four nurses who were working in the units of the hospitals through semi-structured pre-tested questionnaire and checklist. Multi-variable logistic regression was used to analyze the relationship between the dependent and independent variables using odds ratio with a confidence interval of 95% and a p-value of 0.05.

**Results:**

The prevalence of neonatal hypothermia on admission to the neonatal intensive care units in this study area was 50.3%. Admission weight below 2500 gm. (AOR = 3.61, 95% CI: 2.10, 6.18), delay in initiation of breastfeeding (AOR = 2.42, 95% CI: 1.45, 4.02), early bathing (AOR = 2.63, 95% CI: 1.23, 5.63), admissions during cold season (AOR = 1.72, 95% CI: 1.04, 2.84), and presence of obstetrical complication(s) during pregnancy/labor (AOR = 2.46, 95% CI: 1.07, 5.66) were factors significantly associated with hypothermia on admission to the neonatal intensive care units.

**Conclusions:**

The prevalence of neonatal hypothermia on admission to the intensive care units was high. There is a need to create awareness among the community members about the dangers of early bathing and late initiation of breastfeeding. It is also important to give attention to the newborns of mothers with obstetric complications, low-birth-weight babies and babies delivered during the cold season.

## Introduction

The first few minutes afterbirth are the most important time for infant survival as it is the transitional period from intrauterine to extra-uterine life where different adaptations take place[1]. The sudden change in the ambient temperature of the newborns during delivery, especially in the absence of appropriate preventive measures can lead to neonatal hypothermia, defined by the World Health Organization as an axillary temperature of less than 36.5^o^c (97.7°F) [1, 2].

In addition to the sudden change in ambient temperature, newborns are prone to develop hypothermia because of their large surface area per unit of body weight[2]. The problem is more prevalent among very low birth weight and low birth weight babies as they have less subcutaneous fat and a reduced amount of brown fat, and a poorly developed response to thermal stress [2–4]. Lack of attention by health care providers and inappropriate care of newborns during childbirth are additional important factors contributing to neonatal hypothermia[5].

One of the preventive mechanisms of neonatal hypothermia is by maintaining “the warm chain”: warm delivery room, immediate drying, skin-to-skin contact, breastfeeding, postponing weighing and bathing, appropriate clothing and bedding, keeping mother and baby together, warm transportation, warm resuscitation and training and awareness raising[1]. However, those practices are not done routinely at health institutions in less developed countries and are almost absent when the delivery is outside health institutions, all of which increase the risk of neonatal hypothermia in newborns[6].

Neonatal hypothermia is a very common problem throughout the world, including hot tropical countries[6, 7]. The prevalence rate of neonatal hypothermia ranges from 11% - 95% across the world [6–17].

In Ethiopia, very little research has been done with regard to hypothermia in newborns. The dearth of research is especially common in the southern part of Ethiopia, a region where people of different socio-cultural backgrounds live by practicing pastoralist and semi-pastoralist lifestyle. At the moment, the effects of the traditional practices of people from this region which could be potential risk factors for neonatal hypothermia are not known. Further, no published evidence on the risk factors of hypothermia on admission to neonatal intensive care units (NICUs) exists as far as we know. Therefore, the aim of this study was to assess the prevalence and factors associated with neonatal hypothermia on admission to NICUs in Southwest Ethiopia.

## Materials and methods

### Study design

This was an institutions-based cross-sectional study.

### Study area and period

This study was conducted in Arba Minch and Jinka General Hospitals from February to September 2017. The administrative towns of the zones: Arbaminch (Gamo Gofa zone) and Jinka (South Omo zone) are located at a distance of 495 and 735 kilometers southwards respectively from Addis Ababa, the capital of Ethiopia. The average monthly admission rate to Arba Minch General Hospital NICU and Jinka General Hospital NICU is 60 and 40 neonates, respectively. The South Omo zone is one of the zones where maternal and newborn health care services are underutilized as most of the peoples are pastoralists and semi-pastoralists who move from place to place from season to season.

### Source population

All neonates admitted to NICUs of Arba Minch and Jinka General Hospitals

### Study population

Neonates admitted to NICUs of Arba Minch and Jinka General Hospitals during the study period

### Inclusion and exclusion criteria

■ **Inclusion criteria**
  All infants admitted to the NICUs within 28 days of delivery regardless of place of delivery
■ **Exclusion criteria**
  No neonate was excluded from the study

### Sample size determination

A single population proportion formula was used to calculate the minimum sample size required as follows:

$$n=\frac{{\left(Z\alpha /2\right)}^{2}\;*(p)(1-p)}{d^{2}}$$

Where:

n = Minimum sample size required.

p = Estimated prevalence of neonatal hypothermia in newborns (0.70) [18]

d = Maximum tolerable error which is = 0.05

z = Value of standard normal distribution (Z-statistic) at 95% confidence level which is1.96.

$$n=\frac{{\left(1.96\right)}^{2}\;*(0.7)(0.3)}{0.05^{2}}=322.$$

For possible non-response during the data collection time, 10% was added which gave a final sample size of 354.

Because the average monthly admission to the two NICUs is 50, all neonates that were admitted to the NICUs during the study period (February to September) were included in the study.

### Study variables:

■ **Dependent Variable:**
  ➢ Neonatal hypothermia: an axillary temperature of less than 36.5^o^c (97.7°F)
■ **Independent variables**
  ➢ **Physiological factors:** birth weight, age at admission, gestational age at delivery, the presence of birth asphyxia at delivery/need for resuscitation
  ➢ **Behavioral factors:** breastfeeding status, skin-to-skin contact, bathing, use of cap and socks, clothing, mode of delivery
  ➢ **Environmental factors:** room temperature, ambient temperature, time of delivery(daytime vs nighttime)

### Operational definitions

**Delay in initiation of breastfeeding:** Breastfeeding which was not initiated within one hour after delivery

**Early bathing:** Bathing babies before the first 24 hours after birth.

**Hot seasons:** February to May (for months of the data collection only)

**Cold season:** June to September (for months of the data collection only)

**Hypothermia on admission to NICU:** Axillary temperature of less than 36.5^o^c measured during the initial assessment of admission to NICU

### Data collection

A semi-structured questionnaire and checklist were used for the data collection. Data were collected by nurses who were working in the NICUs at the two hospitals. Details of the neonates' history since delivery were collected, including: birth weight, sex, mode of delivery, time of delivery, gestational age at delivery, presence of birth asphyxia/resuscitation, time at which breastfeeding initiated, skin-to-skin contact status, birth attendant, place of delivery, environmental temperature, room temperature, time of delivery and socio-demographic backgrounds of the mothers were collected through face to face interview and by measuring and recording necessary information.

As part of the routine practice at the NICUs, axillary temperature and weight of the neonates were measured and recorded as the neonates arrived to the NICUs. Other anthropometric measurements like head circumference, weight and length of the neonates were also recorded at admission. The weight scales were calibrated weekly. Room temperature reading was recorded daily (for Arba Minch NICU only). Ward head nurse, one supervisor, and the investigators supervised the data collectors closely during the entire data collection period.

### Data quality control

#### Prior to data collection

A pretest was carried out on 5% of neonates on admission to NICU prior to the actual data collection time to check the consistency of the questionnaire and checklist. Data collectors and supervisors were trained for two days on how to collect the data.

#### During data collection

The thermometers were ‘calibrated’ (the measurement had been cross-checked with reference thermometers every week to avoid any false readings due to possible damages to the thermometers).

#### After data collection

The questionnaires and checklists were reviewed and checked for completeness periodically by the principal investigator. Epi-info version 7.1.2.0 was used for data entry to reduce possible errors during data entry. Each and every questionnaire was crosschecked with the entered data and all observed errors were corrected.

### Data processing and analysis

All the questionnaires and the checklists were checked for completeness, coded and entered into Epi Info version 7.1.2.0 and then transferred to SPSS version 24 software for analysis. Descriptive statistics such as mean, percentage, and standard deviation were determined. Bi-variable logistic regression was performed to determine the association between each independent variable and the outcome variable. Variables with p-value less than 0.25 in bi-variable logistic regression were entered to multi-variable logistic regression to adjust the effect of confounders on the outcome variable. The degree of association between dependent and independent variables was determined using the odds ratio with 95% confidence interval. P-value of less than 0.05 was considered significant.

### Ethical consideration

Ethical clearance was obtained from Arba Minch University College of Medicine and Health Sciences Ethical Review Board. An official letter of cooperation was written by the College of Medicine and Health Sciences to the administrators of Arba Minch and Jinka General Hospitals. Written informed consent was obtained from each mother/caregiver of the neonate and each mother/caregiver was informed about the objective of the study and confidentiality of the information she/he was giving. The mothers/caregivers were also informed that participation in the study was voluntary and that she/he had the right to withdraw from participation at any time during the interview.

## Results

### Socio-demographic characteristics of the mothers

Three hundred forty six mothers of neonates admitted to the NICUs of Arba Minch and Jinka general hospitals were interviewed. The mean age of the mothers was 24 years (SD = 4.8). Majority of the mothers, 216(62.4%) were Protestant Christians. Two hundred sixty-nine (77.7%) were from rural areas, 118(34.1%) from Gamo Gofa zone and 248 (71.7%) were housewives. With regard to ethnic group majority, 127 (36.7%) were from Ari, 108 (31.2%) from Gamo and the rest were from 20 different ethnic groups **(Table 1).**

**Table 1:** Socio-demographic characteristics of the mothers of the neonates admitted to Arba Minch and Jinka General Hospital NICUs, Southwest Ethiopia, 2017 (n = 346).

Variable		Frequency	Percent
**Age**	< 20 years	60	17.3
	20–30 years	264	76.3
	> 30 years	22	6.4
**Religion**	Protestant	216	62.4
	Orthodox	103	29.8
	Muslim	10	2.9
	Traditional	17	4.9
**Zone**	Gamo Gofa	118	34.1
	South Omo	213	61.6
	Segen Area	13	3.8
	Other Zones	2	0.5
**Occupation**	House wife	248	71.7
	Private business	24	6.9
	Governmental employee	31	9.0
	Farmer	27	7.8
	Daily laborer/ pastoralist	16	4.6
**Husband’s occupation** **(n = 341)**	Farmer	169	49.6
	Private business	77	22.6
	Governmental employee	74	21.7
	Daily laborers & Pastoralists	21	6.2
**Educational status**	Unable to read & write	129	37.3
	Read & Write	7	2.0
	Elementary school	34	9.8
	Secondary school	70	20.2
	High school/preparatory	64	18.5
	Above grade12	42	12.1
**Husband’s educational status** **(n = 341)**	Unable to read & write	97	28.4
	Elementary school	35	10.3
	Secondary school	79	23.2
	High school/preparatory	85	24.9
	Above grade12	45	13.2
**Mother has her own income**	Yes	125	36.1
	No	221	63.9

### Obstetric characteristics of the mothers

Three hundred thirty-six (97.1%) of the mothers had visited health facilities for antenatal care (ANC) during the recent pregnancy at least one time. Forty-seven (13.6%) of the mothers reported that they had obstetric problems during their most recent pregnancy, with premature rupture of membranes being the most commonly reported problem. Labor was induced in 20 (5.8%) of the mothers. The majority, 207 (59.1%), were hospital deliveries **(Table 2).**

**Table 2:** Obstetrics characteristics of the mothers of the neonates admitted to Arba Minch and Jinka General Hospital NICUs, Southwest Ethiopia, 2017 (n = 346).

Variable		Frequency	Percent
ANC follow up during the last pregnancy	Yes	336	97.1
	No	10	2.9
Number of ANC visits (n = 336)	< 4	155	46.1
	≥4	181	53.9
Obstetric problem during the last pregnancy/labor	Yes	47	13.6
	No	299	86.4
Type of the obstetrical problem (n = 47)	Bleeding	13	27.7
	Hypertension	14	29.8
	Premature rupture of membranes	20	42.5
Onset of labor(n = 341)	Spontaneous	321	94.1
	Induced	20	5.9
Place of birth	Health center	117	33.8
	Home	24	6.9
	Hospital	204	59.0
	In an ambulance	1	0.3
Birth attendant	Family member	15	4.3
	Health professional	322	93.1
	Traditional birth attendant	9	2.6
Parity	Primiparous	155	44.8
	Mutiparous	136	39.3
	Grand multiparous	55	15.9
Labor duration (n = 328)	<12 hours	275	83.8
	12–24 hour	50	15.2
	>24 hours	3	0.9
Type of delivery	Cesarean birth	50	14.5
	Spontaneous vaginal delivery	283	81.8
	Instrumental	13	3.7

### Environmental conditions

Two hundred fifteen (61.1%) of the neonates were admitted to Jinka general hospital and the rest were admitted to Arba Minch General Hospital. More than half of the neonates were born during the daytime, 204 (59.0%) were admitted during daytime and 159 (45.0%) admitted during cold seasons of the year **(Table 3).**

**Table 3:** Environmental conditions during the time of admission of the neonates to the NICUs of Arba Minch and Jinka General Hospitals, Southwest Ethiopia, 2017 (n = 346).

Variable		Frequency	Percent
**Time of delivery**	Day	184	53.2
	Night	162	46.8
**Time of admission**	Day	204	59.0
	Night	142	41.0
**Location of the NICU**	Arba Minch General Hospital	131	37.96
	Jinka General Hospital	215	61.1
**Season of admission**	Cold	159	46.0
	Hot	187	54.0

### Characteristics of the neonates admitted to the NICU

Two hundred five (59.2%) of the neonates admitted to the INCU were males. Breastfeeding problems (49.7%) and neonatal sepsis (41.3%) were the main reasons for admission. Fifty-five (15.9%) of the neonates were bathed within 24 hours after delivery, 176(50.9%) did not breastfeed within the 1^st^ hour after delivery, 108 (31.2%) were not placed on their mother’s abdomen immediately after delivery (no skin-to-skin contact), cap was not used in 327 (94.5%) of the neonates. The median age of the neonates during admission was 12 hours (Interquartile range = 24.5) (**Table 4).**

**Table 4:** Characteristics of the neonates admitted to NICUs in Arba Minch and Jinka general hospitals, Southwest Ethiopia, 2017 (n = 346)).

Variable		Frequency	Percent
Sex	Female	141	40.8
	Male	205	59.2
The main reason for admission (more than one problem in one neonate is possible)	Breast feeding problem	172	49.7
	Neonatal sepsis	143	41.3
	Birth asphyxia	89	25.7
	Prematurity	73	21.1
	Others (Pneumonia & Jaundice)	8	2.4
Baby bathed within 24 hours	Yes	55	15.9
	No	291	84.1
Water used to bath the baby(n = 55)	Cold	9	16.4
	Warm	46	83.6
Baby breastfed within one hour	Yes	170	49.1
	No	176	50.9
Skin-to-skin contact present	Yes	238	68.8
	No	108	31.2
Head covered with cap	Yes	19	5.5
	No	327	94.5
Baby wrapped with dry clothing	Yes	323	93.4
	No	23	6.6
Baby kept apart from mother for >15 minutes	Yes	210	60.7
	No	136	39.3
Baby needed resuscitation to breath	Yes	135	39.0
	No	211	61.0
Any traditional practice done	Yes	0	0
	No	346	100
Baby took water or cattle milk by mouth	Yes	7	2
	No	339	98
Baby’s clothing at the time of admission	Well covered	103	29.8
	Poor	243	70.2
Age of the neonate during admission	≤ 1 hour	68	19.7
	More than 1 hour to 1 day	192	55.5
	More than 1 day to 7 days	81	23.4
	> 7 days	5	1.4

### Prevalence of hypothermia on admission to NICUs

One hundred seventy-four (50.3%) neonates admitted to the NICUs were hypothermic. The prevalence was higher at Jinka General Hospital (58.6%) while it was relatively lower at Arba Minch General Hospital (36.6%).

### Factors associated with neonatal hypothermia on admission to NICU

Delay in initiation of breastfeeding, giving bath within 24 hours after birth, the presence of obstetric problems during pregnancy/labor, baby’s weight less than 2500 gm., and admissions during cold season were factors significantly associated with neonatal hypothermia on admission to NICUs in multivariable analysis. Neonates whose weight was less than 2500gm. on admission were about 4 times more likely to be hypothermic when compared with those with weight greater than or equals to 2500 gm. (AOR = 3.61, 95% CI: 2.10, 6.18). Neonates that did not breastfeed within 1 hour after delivery were about 2.5 times more likely to be hypothermic during admission than those who breastfed (AOR = 2.42, 95% CI: 1.45, 4.02). Neonates that were bathed within 24 hours after delivery were about 2.5 times more likely to develop hypothermia (AOR = 2.63, 95% CI: 1.23, 5.63). Neonates that were admitted during the cold season were about 2 times more likely to be hypothermic on admission than those who were admitted during the hot season (AOR = 1.72, 95% CI: 1.04, 2.84). Neonates born to mothers who had developed obstetric complications during pregnancy/labor were about 2.5 times more likely to be hypothermic during the admission time (AOR = 2.46, 95% CI: 1.07, 5.66)(**Table 5**).

**Table 5:** Bi-variable and multivariable analysis of factors associated with neonatal hypothermia on admission to NICUs of Jinka and Arba Minch General Hospitals, Southwest Ethiopia, 2017 (n = 346).

Variables		Hypothermia		OR (95% CI)	
		Yes	No	COR (95% CI)	AOR (95% CI)
**Weight of the neonate**	<2500gm.	99 (73.3%)	36(26.7%)	**4.99(3.10, 8.01)**	**3.61 (2.10, 6.18)**
	≥2500 gm.	75(35.5%)	136(64.5%)	**1**	1
**Baby breastfed within 1 hour**	No	115(65.3%)	61(34.7%)	**3.54(2.28, 5.52)**	**2.42 (1.45, 4.02)**
	Yes	59(34.7%)	111(65.3%)	**1**	**1**
**Baby bathed within 24 hour**	Yes	42(76.4%)	13(23.6%)	**4.00(2.06, 7.77)**	**2.63 (1.23, 5.63)**
	No	130(44.7%)	161(55.3%)	**1**	**1**
**Season**	Cold	89(56.0%)	70(44.0%)	1.52(1.00, 2.33)	**1.72 (1.04, 2.84)**
	Hot	85(45.5%)	102(54.5%)	1	**1**
**Obstetric complication**	Yes	36(76.6%)	11(23.4%)	**3.82(1.87, 7.76)**	**2.46(1.07, 5.66)**
	No	138(46.2%)	161(53.8%)	1	1
**Residence**	Rural	143(53.2%)	126(46.8%)	1.68(1.91, 2.82)	*
	Urban	31(40.3%)	46(59.7%)	1	
**Time of delivery**	Midnight-morning	57(62.6%)	34(37.4%)	1.68(1.01, 2.83)	*
	Evening-midnight	26(36.6%)	45(63.4%)	0.58 (0.33, 1.03)	
	Daytime	92(50.0%)	92(50.0%)	1	1
**Number of ANC visits**	<4	91(58.7%)	64(41.3%)	1.92 (1.24, 3.00)	*
	≥4	77(42.5%)	104(57.5%)	1	1

* = Not significant in stepwise backward logistic regression. (Wt. of the neonate p- value <0.0001, Baby breast fed: p-value 0.001, Obstetric complication during pregnancy; p-value = 0.035, Season: p -value = 0.034, Baby bathed: p -value = 0.013.

### Outcomes of the neonates admitted to NICUs

Out of the 346 neonates admitted to the two NICUs, 288 (83.2%) survived and were discharged to their homes, while 35 (10.1%) died and the rest 23 (6.6%) were discharged against medical advice (self-discharges). The neonates’ mean length of stay was 7.24 days (SD = 4.23). Hypothermia was associated with increased probability of neonatal death by more than 2-fold (COR = 2.34, 95% CI: 1.11, 4.95) in bi-variable analysis.

## Discussion

In this study, one out of two neonates was hypothermic on admission to the NICUs. This finding is in line with the findings from a study conducted in Brazil between 2010 and 2012 where the prevalence of neonatal hypothermia on admission to NICU was 51%[12]. However, it is lower than the results from studies conducted in Nigeria between 2007 and 2008 where the prevalence of neonatal hypothermia on admission to NICU was 62%[10], a Zimbabwe study in which it was 85%[15] and a study conducted in Ethiopia where the proportion of the neonates with hypothermia at the time of admission to NICU was 64%[19]. The possible reason that the figure is relatively lower than the findings of most the previous studies could be the location of the hospitals as both are in hot climatic zones [10, 11, 15, 19].

Not breastfeeding within the first hour after delivery was one of the factors which were significantly associated with hypothermia on admission to NICU. This is supported by the findings from the studies conducted in Nepal[9], Nigeria[11], Tanzania[15] and northwest Ethiopia[18] where delay in initiation of breastfeeding had increased risk of neonatal hypothermia by 1.5–7.5-fold. This could be due to the fact that breastfed babies get adequate calories from the breast milk which protects them from the hypothermia[20]. Another reason could be those neonates who had breastfed were more likely to be in close or skin-to-skin contact with their mothers’ body which protects them from hypothermia [21, 22]. It could also be that those neonates who did not breastfed were more likely to be given other fluids like cold water or cold sugar water which could have increased their probability of becoming hypothermic[23].

The other factor which has shown significant association with neonatal hypothermia on admission to NICU was the weight of the babies. This is due to the fact that babies with small weight have large surface area per unit of body weight which makes them prone to develop hypothermia[3]. The other reason is, low birth weight babies have decreased thermal insulation due to less subcutaneous fat and the reduced amount of brown fat[2]. This is also supported by the findings from studies conducted in Iran where low birth weight had increased risk of neonatal hypothermia by more than 4-fold, another Iranian study where it had increased risk of hypothermia by about 2.5-fold[14, 24], studies conducted in Nepal[9] and Nigeria[11] where low birth weight had increased risk of neonatal hypothermia by 1.5-fold, and the study conducted in northwest Ethiopia where it had increased the risk by 3.7-fold[18].

The third factor which has shown significant association with neonatal hypothermia on admission to NICU in this study was the season of admission. Similarly, according to research conducted in Nepal, cold season demonstrated increased risk of neonatal hypothermia on admission to NICU by 4-fold[9].

The cold season may cause prolonged exposures to the cold temperatures during which the body begins to loose heat faster than it is produced which will eventually use up the body's stored energy, leading to hypothermia. It may also indicate poor infrastructure and clothing adjustments to the cold season as very few neonates worn cap immediately after delivery, a practice which is stated in the Ethiopian Federal Ministry of Health Guidelines for Integrated Management of Newborn and Childhood to be done routinely.

Another factor which has been associated neonatal hypothermia on admission to NICU in this study is bathing of the babies. This is also supported by one Ugandan study where bathing has increased risk of neonatal hypothermia [25]. The physical effect of the cold water as well as exposing the neonates to the cold environment may explain this. Bathing could also increase risk of hypothermia because a neonate must be separated from the mother’s body contact during the time of bathing.

The fifth factor which has shown association with neonatal hypothermia on admission to NICU in this study is the presence of obstetrical complications like premature rupture of membranes and antepartum hemorrhage during pregnancy and/or labor.

This could be because women who developed obstetrical complications were more likely to need special managements like cesarean section or instrumental deliveries like forceps delivery or the mother might be too sick to be with their neonates in close contact during the time of delivery (skin-to-skin contact with the neonates is less likely to be practiced in such a cases). Some obstetrical complications like premature rupture of membranes might have resulted in neonatal sepsis where fall in body temperature is one of its clinical features.

## Strength and limitations of the study

### The strength of the study

This study included face to face interview which were not carried out by most of the previous studies, making it easier to identify factors such as early bathing of babies.

### Limitations of the study

The main limitation of this study was that it could not address how well certain interventions were performed, such as drying the baby after birth, wrapping with warm/clean clothing during delivery, or quality and duration of skin-to-skin contact, all of which are factors which have high potential to prevent neonatal hypothermia. In addition to this hospital-related factors like the skills and qualifications of the health care providers working in the delivery rooms as well as in the NICUs which might have associated with the outcome of the neonates could not be addressed.

## Conclusion and recommendations

The prevalence of neonatal hypothermia on admission to these two Ethiopian NICUs was high. Delay in initiation of breastfeeding, giving bath within 24 hours of birth, the presence of obstetric problems during pregnancy and/or labor, weight of the neonate less than 2500 gm., and admissions during the cold season were factors significantly associated with neonatal hypothermia on admission to NICUs.

Health workers who are working in the maternal, neonatal and child health units need to adhere to the Ethiopian Federal Ministry of Health Guidelines for Integrated Management of Newborn and Childhood Illness which of course, needs provision of different size caps to delivery rooms to make the care complete. Strengthening health education and counseling about the importance of initiating breastfeeding within the first hour of delivery and delaying bathing babies until 24 hours of delivery is needed.

Giving attention to the neonates of the mothers with complications during pregnancy and/or labor and low birth weight babies is required. Even though the number of neonates who worn cap immediately after delivery was too small to show association with hypothermia, the practice of covering babies with caps and socks should be adopted. Interventional or quasi-experimental studies to address the five areas of significance are recommended.

## Supporting information

S1 File Questionnaire Amharic version. (DOCX)

S2 File Questionnaire English version. (DOCX)

S1 Dataset (SAV)

## Abbreviations

ANC: Antenatal Care
NICU: Neonatal Intensive Care Unit
