# Supplementary material for: Prevalence and factors associated with neonatal hypothermia on admission to neonatal intensive care units in Southwest Ethiopia – A cross-sectional study
Source: PLoS One. 2019 Jun 6;14(6):e0218020. doi: 10.1371/journal.pone.0218020 (PMC6553781; doi:10.1371/journal.pone.0218020)
Supplement: S1 File — (DOCX) [file pone.0218020.s001.docx]

**የፈቃድ ፎርም**

አርባ ምንጭ ዩኒቨርሲቲ የሕክምና እና ጤና ሳይንስ ኮሌጅ

**የጨቅላ ሕፃናት ሰውነት ከመጠን በላይ መቀዝቀዝ/መብረድ ችግር ስፋት፡ ለችግሩ የሚያጋልጡ ሁኔታዎች እንድሁም በቅዝቃዘው ምክንያት የሚከተሉ የጤና ቀውሶችን ለማጥናት በአርባ ምንጭ ዩኒቨርሲቲ ህክምናና ጤና ሳይንስ ኮሌጅ የጥናት ቡድን የተዘጋጀ ቃለ-መጠይቅ፤ ጥቅምት 2017**

ጤና ይስጥልኝ!_______________እባላለሁ፡፡በዚህ ሆስፒታል ከሚሠሩ ሠራተኞች አንዱ ነኝ፡፡ በአሁኑ ጊዜ የጨቅላ ሕፃናት ሰውነት ከመጠን በላይ መቀዝቀዝ/መብረድ ችግርን ለሚያጠኑ የአርባ ምንጭ ዩኒቨርሲቲ ህክምናና ጤና ሳይንስ ኮሌጅ የጥናት ቡድን መረጃ እየሰበሰብኩ ነኝ፡፡ የጥናቱ ዓላማ በአርባ ምንጭ እና በጂንካ ሆስፒታሎች ወደ ጨቅላ ሕፃናት ማቆያ የሚገቡ ጨቅላ ሕፃናት ሰውነት ከመጠን በላይ መቀዝዝ/መብረድ ችግር ስፋት፡ ለችግሩ የሚያጋልጡ ሁኔታዎች እንድሁም በቅዝቃዘው ምክንያት የሚከተሉ የጤና ቀውሶችን ለማጥናት ነው፡፡ ለጥናቱ የሚረዱ መረጃዎችን ከአንቺ እንደማገኝ ተስፋ በማድረግ አንቺን የጥናቱ ተሳታፊ እንድተሆኚ መርጨሻለሁ፡፡ በቃለ-መጠይቁ የመሳተፍና ያለመሳተፍ መብትሽ የተጠበቀ ነው፡፡ እየተሳተፍሽ ካልተመቸሽም በመሃል ማቋረጥ ትችያለሽ፡፡ ጥያቄዎቼን ለመመልስ ጊዜ ካለሽና ፈቃደኛ ከሆንሽ ከ 15 – 20 ደቂቃ የሚፈጁ ጥያቄዎችን ልጠይቅሽ ነኝ፡፡ ስምሽና ሌሎች ስለአንቺ የሚገልፁ መገለጫዎች ስለማይመዘገቡ የሚትሰጪው መረጃ ሚስጥራዊነት የተጠበቀ ይሆናል፡፡ ዛሬ የምትሰጪው መረጃ ወደፊት ከዚህ ጤና ተቋም ከምታገኚው አገልግሎት ጋር በፍጹም ተያያዥነት ስለሌው ምንም ስጋት ልገባሽ አይገባም፡፡እናም በንቃት እና በታማኝነት መሳተፍ ፈቃደኛ ነሽ?

 የምርምሩ ጥቅምና በምርምር ውስጥ የማከናውነውን ሚና ተረድቸ በጥናቱ ለመሳተፍ ተስማምቻለሁ.

*(አይደለም ከሆነ ማቆም፤ አዎን ከሆነ ያስፈርሙና እና ወደ መጠይቁ ይቀጥሉ::)*

አዎ አይደለም

የመረጃ ሰጪዋ ፊርማ ______________

የመረጃ ሰብሳቢዋ ፊርማ ______________

ቀን ____________________________

የተመራማሪዎቹ አድራሻ

1. ገ/ስላሴ ገንድሻ gebretecno@gmail.com ስልክ: 0934596503
2. ከተማ ድሪባ ኢሜል: [ketemadw@gmail.com](mailto:ketemadw@gmail.com) ስልክ 0921777558

ስለፈቃድ ፎርም ዝርዝር መረጃ

አርባ ምንጭ ዩኒቨርሲቲ የሕክምና እና ጤና ሳይንስ ኮሌጅ

**የጨቅላ ሕፃናት ሰውነት ከመጠን በላይ መቀዝቀዝ/መብረድ ችግር ስፋት፡ ለችግሩ የሚያጋልጡ ሁኔታዎች እንድሁም በቅዝቃዘው ምክንያት የሚከተሉ የጤና ቀውሶችን ለማጥናት በአርባ ምንጭ ዩኒቨርሲቲ ህክምናና ጤና ሳይንስ ኮሌጅ የጥናት ቡድን የተዘጋጀ ቃለ-መጠይቅ፤ ጥቅምት 2017**

ይህ የመረጃ ወረቀት እንድትሳተፊበት የምንጠይቀውን የጥናት ፕሮጀክት ለማብራራት የተዘጋጀ ነው፡፡

**የጥናቱ አርዐስት**

**የጨቅላ ሕፃናት ሰውነት ከመጠን በላይ መቀዝቀዝ/መብረድ ችግር ስፋት፡ ለችግሩ የሚያጋልጡ ሁኔታዎች እንድሁም በቅዝቃዘው ምክንያት የሚከተሉ የጤና ቀውሶችን ማጥናት**

**የዋና ተመራማሪ ስም፡-** ገ/ብረስላሴ ገንድሻ

**የድርጅቱ ስም፡-** አርባ ምንጭ ዩኒቨርሲቲ, የህክምና እና የጤና ሳይንስ ኮሌጅ

**የስፖንሰር አድራጊው ስም፡-** አርባ ምንጭ ዩኒቨርሲቲ

**የጥናቱ ፕሮጀክት ዓላማ**

የዚህ የምርምር ፕሮጀክት ዋና ዓላማ የጨቅላ ሕፃናት ሰውነት ከመጠን በላይ መቀዝቀዝ/መብረድ ችግር ስፋት፡ ለችግሩ የሚያጋልጡ ሁኔታዎች እንድሁም በቅዝቃዘው ምክንያት የሚከተሉ የጤና ቀውሶችን ማጥናት ነው፡፡ የጨቅላ ሕፃናት ሰውነት ከመጠን በላይ መቀዝቀዝ/መብረድ ችግር ስፋት፡ ለችግሩ የሚያጋልጡ ሁኔታዎች እንድሁም በቅዝቃዘው ምክንያት የሚከተሉ የጤና ቀውሶችን ማጥናት ለወደፊት ለችግሩ መፍትሔ ለማበጀት መንገድ ጠራጊ እርምጃ ነው፡፡

**ሂደት**

ከአርባ ምንጭ ዩኒቨርስቲ ምርምሩን ለማካሄድ ፊቃድ አግኝተናል፡፡ወደ አርባ ምንጭ እና ጅንካ አጠቃላይ ሆስፒታሎችም ትብብር ደብዳቤ ተጽፏል፡፡ጥናቱ በአርባ ምንጭ እና ጅንካ ሆስፒታሎች የጨቅላ ሕጻናት ህክምና ክፍል የተኙ ሕጻናትን በሙሉ ያካትትታል፡፡ ስለዚህ እርስዎ በዚህ ጥናት ለመሳተፍ ፍቃደኛ ከሆኑ እንዲሳተፉ ተመርጠዋል፡፡

ለመሳተፍ ፈቃደኛ ከሆኑ በጣም ደስ ይለናል፡፡ እናም የዚህን ጥናት አላማ በትክክል መረዳትዎን እና ስምምነትዎን ማየት እንፈልጋለን፡፡ በመጨረሻም ትክክለኛውን ምላሽዎን ብቻ እንዲሰጡ በአክብሮት እነንጠይቅዎታለን፡፡

**ጉዳት**

በዚህ ምላሽ ላይ በመሳተፍ ከምንወስድብዎት ጊዜ ውጭ (ለ 20 ደቂቃዎች) በምላሽ በመሳተፍ የሚያጋጥምዎት ምንም ዓይነት ጉዳት ወይም ምቾት ማጣት አይኖርም፡፡ በምዝገባ መጽሐፎዎች ውስጥ የተመዘገበ ማንኛውም የግል መረጃ አይወሰድም፡ ወደ ሌላ አካል አይዛወርም፡፡ እያንዳንዱ መረጃ በምስጢር ይቀመጣል፡፡

**ጥቅም**

በዚህ ጥናት መሳተፍሽ የጨቅላ ሕፃናት ሰውነት ከመጠን በላይ መቀዝቀዝ/መብረድ ችግር ስፋት፡ ለችግሩ የሚያጋልጡ ሁኔታዎች እንድሁም በቅዝቃዘው ምክንያት የሚከተሉ የጤና ቀውሶችን ለማወቅ በጣምይረዳናል፡፡ በጥናቱ ውጤት ላይ በመመርኮዝም ለወደፊት መፍትሔዎች ይዘጋጃሉ፡፡ ይሁን እንጂ በዚህ የምርምር ፕሮጀክት ላይ በመሳተፍሽ ምንም አይነት ጉዳት ወይም ቀጥተኛ ጥቅም አታገኝም፡፡

**ለመሳተፍ ማበረታቻዎች / ክፍያዎች**

በዚህ ፕሮጀክት ለመሳተፍ ማበረታቻ ወይም ክፍያ አይሰጥዎትም፡፡

**ሚስጢራዊነት**

ከርስዎ የተሰበሰበ መረጃ ስምሽ ሳይጠቀስ በምስጢር ይቀመጣል፡፡ በስምሽ ፋንታ በምስጢር ውስጥ ያለ የኮምፒተር ቁጥር ስለምሰጥ የእርስዎ ማንነት የሚገልጽ ነገር አይኖርም፡፡

**የመተው ወይም የማቋረጥ መብት**

በዚህ ምርምር ውስጥ ላለመሳተፍ ሙሉ መብት አለዎት፡፡ ከፈለጉ መጠይቁን ከጀመሩ በኋላ መሀል ላይ ማቋረጥም ይችላሉ፡፡

የተመራማሪዎቹ አድራሻ

ይህ የምርምር ፕሮጀክት በአርባ ምንጭ ዩኒቨርስቲ የምርምር ስነ-ምግባር ኮሚቴ ፀድቋል፡፡ ማንኛውም ጥያቄ ቢኖርዎት የሚከተለውን ግለሰብ ማነጋገር ይችላሉ ፤ እንዲሁም በማንኛውም ጊዜ መጠየቅ ይችላሉ.

1. ገ/ስላሴ ገንድሻ gebretecno@gmail.com ስልክ: 0934596503
2. ከተማ ድሪባ ኢሜል: [ketemadw@gmail.com](mailto:wanzanati2011@gmail.com) ስልክ 0921777558

**ክፍል 1. አጠቃላይ መረጃ**

| ተ.ቁ | ጥያቄ | መልስ | ምርመራ |
| --- | --- | --- | --- |
|  | ህፃኑ የተኛበት/የገባበት ቀን | ________/_____/2017 |  |
|  | የገባበት ሰዓት | : |  |
|  | የሆስፒታሉ ስም | 1. ጂንካ 2. አርባ ምንጭ |  |
|  | የቴርሞሜትሩ ዓይነት | 1. ድጅታል 2. ስኬል/ሜርኩሪ |  |
|  | ክፍሉ ቴርሞሜተር አለው | 1. አዎን 2. አይደለም |  |
|  | ካለው የዛሬው ቀን የክፍሉ አማካይ ሙቀት ስነት ነው; | ________^o^ሴ. |  |
|  | ሙቀት የሚለካበት መንገድ | 1. ብብት ስር 2. በአፍ 3. በፊንጢጣ 4. በጆሮ 5. በጉሮሮ |  |

**ክፍል 2 የእናት ማህበራዊ ኩነቶች**

| ተ.ቁ | ጥያቄ | መልስ | ምርመራ |
| --- | --- | --- | --- |
|  | የዚህ ልጅ እናት ነዎት | 1.አዎን  2.አይደለም |  |
|  | ከየትኛው ዞን ነው የመጣችሁት? | 1. ጋሞ ጎፋ 3. ደቡብ ኦሞ 2. ሰገን አካባቢ 4.ሌላ__________ |  |
|  | ከየትኛው ወረዳ? |  |  |
|  | ዕድሜዎት ስንት ዓመት ነው/የእናት/ | __________ዓመት |  |
|  | ሥራዎ ምንድነው? /የእናት/ | 1. የቤት እመቤት 4. የግል ሥራ/ንግድ/ 2. የመንግስት ሠራተኛ 5. አርሶ አደር 3. አርብቶ ደር 6. ሌላ(ይገለፅ) |  |
|  | የትዳር ሁኔታዎ? /የእናት/ | 1. ያገባች 3. ያላገበች 2. የተፋታች 4. ባል የሞተባት | ያላገባች ከሆነ ወደ ቁ.8 ይለፉ |
|  | የባለቤትዎ ሥራ ምንድነው? /የእናት/ | 1. የመንግስት ሠራተኛ 4. አርብቶ ደር 2. የግል ሥራ/ንግድ 5. አርሶ አደር 3. ሌላ (ይገለፅ)____________ |  |
|  | የየትኛው ብሔር ተወላጅ ነዎት? /የእናት/ |  |  |
|  | የየትኛው ሀይማኖት ተከታይ ነዎት? /የእናት/ | 1. ኦርቶዶክስ 2. ሙስሊም 2. ፕሮቴስታንት 3. ሌላ(ይገለፅ______________ |  |
|  | የትምህርት ደረጃዎስ?/የእናት/ | 1. ያልተማረች 2. መፃፍና ማንበብ የሚትችል 3. አንደኛ ደረጃ (ከ1ኛ -4ኛ ክፍል) 4. ሁለተኛ ደረጃ(ከ5ኛ-8ኛ ክፍል) 5. ከፍተኛ/መሰናዶ.(ከ9ኛ -12ኛ ክፍል) 6. ከ12ኛ ክፍል በላይ |  |
|  | የትዳር ጓደኛዎ የትምህርት ደረጃስ?/የእናት/ | 1. ያልተማረ 2. መፃፍና ማንበብ የሚችል 3. አንደኛ ደረጃ (ከ1ኛ -4ኛ ክፍል) 4. ሁለተኛ ደረጃ(ከ5ኛ-8ኛ ክፍል) 5. ከፍተኛ/መሰናዶ.(ከ9ኛ -12ኛ ክፍል) 6. ከ12ኛ ክፍል በላይ | ካላገባች NA በል/ይ |
|  | የራስሽ የሆነ የወር ገቢ አለሽ | 1 አዎን 2. አይደለም | አይደለም ከሆነ ወደ ቁ. 14 ይለፉ |
|  | ካለሽ የወር ገቢሽ ምን ያህል ይሆናል?/የእናት/ | ___________ ብር |  |
|  | የባለቤትዎ የወር ገቢ ምን ያህል ብር ይሆናል?  /የእናት/ | ___________ ብር | ባለትዳር ካለሆነች NA |
|  | የት ነው የሚኖሩት/የእናት/ | 1. ከተማ 2. ገጠር |  |

**ክፍል 3. የሥነ-ወሊድ ታሪክ** /**የእናት**/

| ተ.ቁ | ጥያቄ | መልስ | ምርመራ |
| --- | --- | --- | --- |
|  | በአጠቃላይ ስንት ጊዜ ነው ያረገዙት? | _______________ ጊዜ |  |
|  | በአጠቃላይ ስንት ጊዜ ነው የወለዱት? | _____________ ጊዜ |  |
|  | ለመጨረሻው እረግዝና የቅድሜ ወሊድ ክትትል አድርገው ነበር? | 1. አዎን 2. አይደለም | አይደለም ከሆነ ወደ ቁ.5 ይለፉ |
|  | ክትትል ታደረጊ ከነበረ ስንት ጊዜ ነው የሄድሽው? | ________ ጊዜ |  |
|  | ባለሙያዎቹ በእርግዝና ክትትሉ ጊዜ /በእርግዝናሽ ላይ ችግር አለ ብለው ነግረውሽ ያውቃሉ? | 1.አዎን  2. አይደለም | አይደለም ከሆነ ወደ ቁ.7 ይለፉ |
|  | አዎን ከሆነ ምንድነው? | 1. የደም ግፊት መጨመር 2. የደም መፍሰስ 3. የስኳር በሽታ 4. የሽረት ውሃ ያለ ጊዜ መፍሰስ 5. ሌላ (ይገለፅ)________ |  |
|  | በምጥ ላይ ምን ያህ ሰዓት ነው የቆየሽው? | _______________ሰዓት | በC/S ከወለደች NA |
|  | ምጥሽ የጀመርሽ በራሱ ጊዜ ነው ወይስ መድሃኒት ተሰጥቶሽ ነው? | 1. በራሱ ጊዜ 5. NA 2. በመድኃኒት 3. አላወቅሁም/ | በC/S ከወለደች NA |

**ስለጨላ ሕጻኑ ሁኔታ**

|  | ፈስዮሎጂካል ፋክተሮች | | |
| --- | --- | --- | --- |
| ተ.ቁ | ጥያቄ | መልስ | ምርመራ |
|  | ፆታ | 1. ወንድ 2. ሴት |  |
|  | የህጻኑ ክብደት በግራም ወደ ክፍሉ ሲገባ | _____ ግ. |  |
|  | ስወለድ የነበረው ክብደት በግራም | _______ግ. | ከሌለNA በል |
|  | Gestational age at delivery in days (የተመዘገበ ነገር ካለ ወይንም እናትን LMP ይጠይቁ ካስታወሰች) | 1. ____ቀናት 2. NA | NA በል LMP ካላወቀች |
|  | የሕጻኑ ዕድሜ በሰኣት (ከተወለደ ጀምሮ) | _____ ሰዓት. |  |
|  | ልጁ ወደዚህ የገባባት ዋና ምክኒያት | 1. Hypothermia 2. Birth asphyxia 3. Prematurity 4. Jaundice 5. Sepsis 6. Pneumonia 7. LBW 8. Breast feeding problem 9. Other _________ |  |

|  | ሌሎች ሁኔታዎች | | |
| --- | --- | --- | --- |
| ተ.ቁ | ጥያቄ | መልስ | ምርመራ |
|  | ልጁ የተወለደበት ቦታ | 1. ቤት 2. ጤና ኬላ 3. ጤና ጣቢያ 4. ሆስፒታል 5. በአምቡንስ ውስጥ 6. በራሴ |  |
|  | ማነው ያዋለደሽ | 1. የቤተሰብ አባል 2. የልምድ ኣዋላጅ 3. የጤና ኤክስተንሽን ባለሙያ 4. የጤና ባለሙያ 5. ሌላ ______________ |  |
|  | የወለድሽው በማህጸን ነው ወይስ በኦፕራሲዮን/ በመሳሪያ በመታገዝ ነው? | 1. SVD 2. C/S 3. Forceps delivery 4. Vacuum delivery | በመሳሪያ ነው ካለች ምን ዓይነት እደሆነ በደንብ ጠይቁ |
|  | ልጁ በተወለደ በ24 ሰዓት ጊዜ ውስጥ ታጥቦ ነበር? | 1. አዎን 2. አይደለም | አይደለም ከሆነ ወደ ቁ. 6 ይለፉ |
|  | ከታጠበ በምን ዓይነት ውሃ ነው ? | 1. ለብ ያለ ውሃ 2. ቀዝቃዛ ውሃ 3. ሌላ ፈሳሽ   (ይገለፅ)____ |  |
|  | ልጁ በተወለደ በ1 ሰዓት ውስጥ ጡት ጠብቶ ነበር? | 1. አዎን 2. አይደለም | አይደለም ከሆነ ወደ ቁ. 8 ይለፉ |
|  | ከጠባ የመጀመሪያው ወተት /እንገር/ ከላይ ከፈሰሰ በኋላ ነው የጠባው? | 1. አዎን 2. አይደለም |  |
|  | ልጁ እንደተወለደ ከእናቱ ጋር የቆዳ ለቆዳ ንክክ አድረጎ ነበር/እንድሞቀው/? | 1. አዎን 2. አይደለም |  |
|  | ልጁ ከተወለደ በኋላ ኮፊያ ተደርጎለት ነበር? | 1. አዎን 2. አይደለም |  |
|  | ልጁ ከተውለደ በኋላ ካልሲ ተደርጎለት ነበር | 1. አዎን 2. አይደለም |  |
|  | ልጁ እንደተወለደ በመጀመሪያው ደቂቃ ውስጥ በደንብ እንድደርቅ ተደርጎ ነበር ? | 1. አዎን 2. አይደለም 3. አላወቅሁም |  |
|  | ልጁ በደረቅ ጨርቅ ተጠቅልሎ ነበር ? | 1. አዎን 2. አይደለም 3. አላወቅሁም |  |
|  | ሕፃኑ ከተወለደ በኋላ ከ15 ደቂቃ በላይ ለሆነ ጊዜ ከእናቱ ተለይቶ ነበር? | 1. አዎን 2. አይደለም |  |
|  | ሕፃኑ ከተወለደ በራሱ መተንፈስ አቅቶት ከባለሙያዎች እገዛ በኋላ ጀምሮ ነበር? | 1. አዎን 2. አይደለም |  |
|  | በባህላችሁ ሕፃን ልጅ እንደተወለደ የሚደረግበት ድርጊት አለ እንደ በዚህ ሕፃን ላይም የተደረገ? | 1. አዎን 2. አይደለም 3. አላውቅም | አዎን ካልሆነ ወደ ቁ. 17 ይለፉ |
|  | ከሆነ ምንድነው? | __________________ |  |
|  | ሕፃኑ በአፉ የተሰጠው ነገር አለ እንዴ ? | 1. አዎን 2. አይደለም | አይደለም ከሆነ ወደ ቁ. 19 ይለፉ |
|  | ከሆነ ምንድነው? | 1. ዉሃ 2. ቅቤ 3. ዘይት 4. ወተት 5. ሌላ (ይገለፅ)______ |  |
|  | ልጁ ወደዚህ ሲመጣ እንደት ነበር? | 1. በደንብ ኮፊያና ልበስ ተደርቦለት ከእናት/ተንባካቢ/ጋር የቆዳ ለቆዳ ንክክ የነበረበት ጥሩ አያያዝ 2. በደንብ ኮፊያና ልበስ ተደርቦለት ግን ከእናት/ተንባካቢ/ጋር የቆዳ ለቆዳ ንክክ ያልነበረበት አያያዝ 3. ቀለል ባለ ልበስ ብቻ ነው የተሸፈነው 4. ሌላ _______________ |  |

|  | የአከባቢ ሁነታዎች | | |
| --- | --- | --- | --- |
| ተ.ቁ | ጥያቄ | መልስ | ምርመራ |
|  | ልጁ የተወለደበት ጊዜ? | 1. ማታ_______፡________ ሰኣት 2. ቀን ________፡_______ሰዓት | (12:00– 12:00) |
|  | ልጁ የተወለደበት ቀን አየሩ ምን ይመስል ነበር? | 1. ሞቃታማ 2. መካከለኛ 3. ቀዝቃዛ |  |

አመሰግናለሁ

|  | Checklist for the Outcome of the neonate | | | | |
| --- | --- | --- | --- | --- | --- |
| SN | Question | Response | | | Remarks |
|  | What was the final outcome of the neonate | 1. Cured and discharged 2. Died 3. Discharged against medical advise | | |  |
|  | How long was the neonate stayed in the NICU | _________ days | | |  |
|  | Did the baby develop any of the following complication during the course of treatment? (Check the medical chart and thick under appropriate column) | Yes | No | NA(Not measured) |  |
|  | 1. Respiratory distress |  |  |  |  |
|  | 1. Metabolic acidosis |  |  |  |  |
|  | 1. Pulmonary edema |  |  |  |  |
|  | 1. DIC |  |  |  |  |
|  | 1. Jaundice |  |  |  |  |
|  | 1. Hypoglycemia |  |  |  |  |
|  | 1. Other ____________________________________________ |  |  |  |  |
