# Supplementary material for: Prevalence and factors associated with neonatal hypothermia on admission to neonatal intensive care units in Southwest Ethiopia – A cross-sectional study
Source: PLoS One. 2019 Jun 6;14(6):e0218020. doi: 10.1371/journal.pone.0218020 (PMC6553781; doi:10.1371/journal.pone.0218020)
Supplement: S2 File — (DOCX) [file pone.0218020.s002.docx]

## CONSENT FORM

## Arba Minch University College of Medicine and Health Sciences

## Consent form for a research project with a title Neonatal hypothermia on admission to neonatal intensive care units of Arba Minch and Jinka general hospitals

**Hello**!

My name is _______________. I am working for investigators from Arba Minch University who are doing a research on neonatal hypothermia on admission to neonatal intensive care units (NICU) of Arba Minch and Jinka general hospitals. I will ask you few questions about your socio-demographic characteristics and your baby’s history which may take 15 – 20 minutes for you to respond. We will use anthropometric measurements of your baby in the research too. This will help us identify factors associated with neonatal hypothermia based on your answer to our questions. Mothers/care takers of all the neonates admitted to this NICU are asked and you are one of them to participate in the assessment.

The goal of this study is to generate information on neonatal hypothermia, and associated factors with hypothermia during admission to this NICU which may help concerned bodies responsible and authorities such as regional hearth bureau, zonal health departments, & others to take actions (interventions) based on the study findings. To effectively reach at the predetermined goal of the research, I am requesting your help. The questionnaire do not concern on the private (personal) information, which will maintain confidentiality. You have full right to refuse part of or whole of the questionnaires & no one in force you to do so. However your honest participation and answers to the questions will help us in better understanding of the problem and to give guidance on how to intervene within the study area. So are you willing to participate actively and honestly?

I understood about the advantage of the research, the roles I will have in the research and have agreed to participate in the research. (*If yes, let her sign and go ahead, if No stop here*.)

Yes Signature of the participant _____________ No

Signature of the data collector __________________________

Date: ______________________________________________

Contact persons:

1. Gebresilasea Gendisha Email: [gebretecno@gmail.com](mailto:gebretecno@gmail.com) Phone: 0934596503
2. Ktema Diriba Email: [ketemadw@gmail.com](mailto:ketemadw@gmail.com) Phone: 0921777558

## INFORMATION SHEET

## Arba Minch University College of Medicine and Health Sciences

## Information Sheet for a research project with a title neonatal hypothermia on admission to neonatal intensive care units of Arba Minch and Jinka general hospitals

This information sheet is prepared to explain the research project that you are asked to join by a group of research investigators.

**Title of the Research Project**

Neonatal hypothermia on admission to neonatal intensive care units of Arba Minch and Jinka general hospitals

Name of the Principal Investigator: Gebresilasea Gendisha

**Name of the organization:** Arba Minch University, College of Medicine and Health Sciences

**Name of the sponsor:** Arba Minch University

**Purpose of the research project**

The main aim of this research project is to assess the prevalence and factors associated with neonatal hypothermia on admission to neonatal intensive care units of Arba Minch and Jinka general hospitals.

Assessing factors associated with neonatal hypothermia is important to reduce neonatal morbidity and mortality. Because the results of this study will be used to design appropriate intervention programs to address the problem in the study area.

**Procedure**

Letter of permission was obtained from Arba Minch University to Arba Minch and Jinka General Hospitals. The study involves mothers/caregivers of neonates admitted to the two hospitals NICU. You are selected to be one of the study participants if you are willing to take part in this study and we kindly invite you to take part in our project.

If you are willing to participate, we are so happy and we need you to clearly understand the aim of this study and show your agreement .Finally you are kindly requested to give your genuine response.

**Risk and/or discomfort**:

There is no any risk or discomfort that you will face by participating in this research except dedication of time (a maximum of 20 minutes) for responding. Any personal information registered in registration books will not be copied and transferred to other bodies. Every piece of information will be kept confidential.

**Benefits**

Your participation is definitely important to identify factors associated with neonatal hypothermia and to design appropriate strategy to decrease the incidence of neonatal hypothermia as well as its poor sequels. The findings of this study are used to implement the intervention and reveal out the problem related to neonatal hypothermia and provides information on factors associated with it. There is no risk or direct benefit in participating in this research project.

**Incentives/payments for participating**

You will not be provided with any incentives or payment to take part in this project.

**Confidentiality**

The information collected from you will be kept confidential and stored in a file, without your name by assigning a code number to it. And hence no report of the study ever identifies you.

**Right to refuse or withdraw**

You have a full right to refuse from participating in this research. You have also a full right to withdraw from this study at any time you wish.

**Person to contact**

This research project was reviewed and approved by the ethical committee of Arba Minch University. If you have any question you can contact the following individual and you may ask at any time you want.

Name: Gebresilasea Gendisha [gebretecno@gmail.com](mailto:gebretecno@gmail.com) Phone 09 34 59 65 03 Or Ketema Diriba Email: [ketemadw@gmail.com](mailto:ketemadw@gmail.com) Phone 0921777558

## QUESTIONNAIRE (ENGLISH VERSION)

**Arba Minch University College of Medicine and Health Sciences**

Questionnaire to assess prevalence and factors associated with neonatal hypothermia on admission to NICUs, of Arba Minch and Jinka general hospitals.

**Part I. General Information about the health institutions**

| **SN** | **Question** | **Answer** | **Remarks** |
| --- | --- | --- | --- |
|  | Date of admission | DD______/MM____/2017 |  |
|  | Time of admission |  |  |
|  | Name of the hospital | 1. Arba Minch General Hospital 2. Jinka General Hospital |  |
|  | Type of thermometer you are using | 1. Digital 2. Scale |  |
|  | Is there room thermometer in the NICU | 1. Yes 2. No |  |
|  | If present what is the average temp the NICU room today | ________^o^c. |  |
|  | Route of temp measurement | 1. Axillary 2. Oral 3. Rectal 4. Tympanic 5. Esophageal |  |

**Part-I socio demographic characteristics of the Mother**

| **SN** | **Question** | **Response** | **Remarks** |
| --- | --- | --- | --- |
| 1. . | Are you the mother of this baby? | **1**.Yes  2.No |  |
|  | From which zone did you come? | 1. Gamo Gofa Zone 2. Segen area Peoples zone 3. South Omo zone 4. Other zone Specify**________________** |  |
|  | What is the name of District you came from? | ___________________ |  |
|  | What is your age in completed years | _______________ years |  |
|  | What is your occupation? | 1. House wife 2. Government employee 3. Private business 4. Farmer 5. Pastoralist 6. Other (Specify) ______________ |  |
|  | What is your marital status? | 1. Married 2. Divorced 3. Single 4. Widowed 5. Separated | Go to Q # 8 if not married |
|  | What is the occupation of your husband  ? | 1. Government employee 2. Private business 3. Farmer 4. Pastoralist 5. Other (Specify)__________ |  |
|  | To which ethnic group do you belong? | ________________________ |  |
|  | To which religious groups do you belong? | 1. Orthodox 2. Protestant 3. Muslim 4. Other (Specify)______________ |  |
|  | What is the highest grade you completed? | 1. Unable to read & write 2. Read and write 3. Elementary school (grade 1 -4) 4. Secondary school(Grade 5-8) 5. High school/prep.(grade 9 -12) 6. Above grade 12 | Go to Q # 12 if not married |
|  | What is the educational level of your husband? | 1. Unable to read & write 2. Read and write 3. Elementary school (grade 1 -4) 4. Secondary school(Grade 5-8) 5. High school/prep.(grade 9 -12) 6. Above grade 12 |  |
|  | Do you have your own income? | 1.Yes  2.No | If no go to Q#14 |
|  | What is your monthly income level in ETB? | __________ ETB |  |
|  | What is monthly income level of your husband? | ___________ ETB | Write NA if not married |
|  | Where are you living now? (Residence) | 1. Urban area 2. Rural area |  |

**Part III Obstetric Characteristics**

| **SN** | **Question** | **Response** | **Remarks** |
| --- | --- | --- | --- |
|  | How many times did you get pregnant? (Gravidity) | _______________ times |  |
|  | How many times did you give birth? (Parity) | _____________ times |  |
|  | Do you have ANC follow up during the most recent pregnancy? | 1. Yes 2. No | Go to Q # 5 if NO |
|  | If yes, how many times did you visit the ANC clinic? | ________ times |  |
|  | Have you ever told by your ANC provider that there was problem with your pregnancy? | 1. Yes 2. No | Go to Q # 7 if NO |
|  | If yes, what was the problem | 1. Hypertension 2. Bleeding 3. DM 4. PROM 5. Other (Specify)_______ |  |
|  | Can you tell me the total duration of your labor in hours? | _______________ hours | NA if elective C/S |
|  | Was your labor started spontaneously or induced? | 1. Spontaneous 4/ NA 2. Induced 3. I do not know | NA if elective C/S |

**Part IV Respe**

**Part INeon**

**Neonate related questions**

|  | **Physiologic factors** | | |
| --- | --- | --- | --- |
| **SN** | **Question** | **Response** | **Remarks** |
|  | Sex of the neonate | 1. Male 2. Female |  |
|  | Weight of the baby in grams (on admission) | _____ grms |  |
|  | Birth Wt if available (in grams) | _____gms | If no, NA |
|  | Gestational age at delivery in days (if any record, or ask the mother if she remember LMP) | 1. ____days 2. NA | NA if LMP is unknown |
|  | Age of the neonate after delivery in hours | _____ hrs. |  |
|  | The main diagnosis at admission | 1. Hypothermia 2. Birth asphyxia 3. Prematurity 4. Jaundice 5. Sepsis 6. Pneumonia 7. LBW 8. Breast feeding problem 9. Other _________ |  |

|  | **Behavioral factors** | | |
| --- | --- | --- | --- |
| **SN** | **Question** | **Response** | **Remarks** |
|  | Place of delivery | 1. Home 2. Health post 3. Health center 4. Hospital 5. In ambulance 6. Other (Specify)________ |  |
|  | Who assisted you during the birth of this baby? | 1. Family member /relative 2. Traditional birth attendant 3. Health Extension worker 4. Health professional 5. No one (by myself) |  |
|  | Type of delivery | 1. SVD 2. C/S 3. Forceps delivery 4. Vacuum delivery | If instrumental delivery, ask the mother about what does the instrument looks like |
|  | Was the baby bathed within 24hours after delivery? | 1. Yes 2. No |  |
|  | Type of water used to bath the baby | 1. Warm water 2. Cold water 3. Other fluid (specify)______ |  |
|  | Was the baby breast fed within 1 hour after delivery? | 1. Yes 2. No | If No, go to Q # 8 |
|  | If yes, was colostrum extracted? | 1. Yes 2. No |  |
|  | Was the baby delivered on the mother’s bare abdomen (skin to-skin-contact) present? | 1. Yes 2. No |  |
|  | Was the baby’s head covered with cap immediately after delivery? | 1. Yes 2. No |  |
|  | Was the baby wore socks immediately after delivery | 1. Yes 2. No |  |
|  | Was the baby thoroughly dried with clean cloth within the first minute after delivery? | 1. Yes 2. No |  |
|  | Was the warped by a dry clean cloth after dried thoroughly? | 1. Yes 2. No |  |
|  | Was the baby separated from you for longer period (>15 minutes) | 1. Yes 2. No |  |
|  | Was the baby needed mechanical ventilation to start berating /resuscitated?? | 1. Yes 2. No |  |
|  | Is there any traditional custom which is practiced on newborns in your community which was applied on this neonate after delivery? | 1. Yes 2. No 3. I do not know | If No, go to Q # 17 |
|  | If yes, what? | __________________  __________________ |  |
|  | Was the neonate given anything by mouth yet? | 1. Yes 2. No | If No, go to Q # 19 |
|  | If yes, what? | 1. Water 2. Better 3. Oil 4. Milk 5. Other (specify)______ |  |
|  | How was the baby transported to the NICU? | 1. Well covered with cape and warm clothes and in skin-to skin contact with caregiver/mother 2. Head covered with hat baby well covered with thick clothing but not in skin to skin contact with mother/caretaker 3. Covered with think clothing only 4. Other _______________ |  |

|  | **Environmental Factors** | | |
| --- | --- | --- | --- |
| **SN** | **Question** | **Response** | **Remarks** |
|  | At what time of the baby born? | 1. Night time 2. Day time | (12:00– 12:00) |
|  | Environmental temperature of the day of delivery | 1. Hot 2. Warm 3. Cold | Subjective |

**Thank you very much.**

|  | **Checklist for the Outcome of the neonate** | | | | |
| --- | --- | --- | --- | --- | --- |
| **SN** | **Question** | **Response** | | | **Remarks** |
|  | What was the final outcome of the neonate | 1. Cured and discharged 2. Died 3. Discharged against medical advise | | |  |
|  | Did the baby develop any of the following complication during the course of treatment? | **Yes** | **No** | **NA(Not measured)** | Check the medical chart and thick under appropriate column |
|  | 1. Respiratory distress |  |  |  |  |
|  | 1. Metabolic acidosis |  |  |  |  |
|  | 1. Pulmonary edema |  |  |  |  |
|  | 1. DIC |  |  |  |  |
|  | 1. Jaundice |  |  |  |  |
|  | 1. Hypoglycemia |  |  |  |  |
|  | 1. Others (list) |  |  |  |  |
